# Autocrine Role of Angiopoietins during Megakaryocytic Differentiation

**DOI:** 10.1371/journal.pone.0039796

**Published:** 2012-07-06

**Authors:** Ernestina Saulle, Raffaella Guerriero, Alessia Petronelli, Elena Coppotelli, Marco Gabbianelli, Ornella Morsilli, Isabella Spinello, Elvira Pelosi, Germana Castelli, Ugo Testa, Simona Coppola

**Affiliations:** 1 Department of Hematology, Oncology and Molecular Medicine, Istituto Superiore di Sanità, Rome, Italy; 2 National Center for Rare Diseases, Istituto Superiore di Sanità, Rome, Italy; Ospedale Pediatrico Bambino Gesu’, Italy

## Abstract

The tyrosine kinase Tie-2 and its ligands Angiopoietins (Angs) transduce critical signals for angiogenesis in endothelial cells. This receptor and Ang-1 are coexpressed in hematopoietic stem cells and in a subset of megakaryocytes, though a possible role of angiopoietins in megakaryocytic differentiation/proliferation remains to be demonstrated. To investigate a possible effect of Ang-1/Ang-2 on megakaryocytic proliferation/differentiation we have used both normal CD34^+^ cells induced to megakaryocytic differentiation and the UT7 cells engineered to express the thrombopoietin receptor (TPOR, also known as c-mpl, UT7/mpl). Our results indicate that Ang-1/Ang-2 may have a role in megakaryopoiesis. Particularly, Ang-2 is predominantly produced and released by immature normal megakaryocytic cells and by undifferentiated UT7/mpl cells and slightly stimulated TPO-induced cell proliferation. Ang-1 production is markedly induced during megakaryocytic differentiation/maturation and potentiated TPO-driven megakaryocytic differentiation. Blocking endogenously released angiopoietins partially inhibited megakaryocytic differentiation, particularly for that concerns the process of polyploidization. According to these data it is suggested that an autocrine angiopoietin/Tie-2 loop controls megakaryocytic proliferation and differentiation.

## Introduction

Angiopoietins are a family of molecules known to bind to, and activate, the Tie (Tyr kinase with Ig and EGF homology domains) receptors, Tie-1 and Tie-2 receptor on endothelial cells [1]. Tie-1 and Tie-2 receptors have a unique structure containing extracellular epidermal growth factor homology domains, Ig-like loops, and fibronectin type III homology domains [2], [3]. Angiopoietins play a key role in the regulation of angiogenesis and vascular homeostasis. Angiopoietin-1 (Ang-1) is required for the maintenance of the integrity of endothelium, whereas Angiopoietin-2 (Ang-2) was considered to act as an antagonist, destabilizing the vasculature [1]. However, recent evidences, suggest that the effect of Ang-2 is dependent on the local cytokine milieu: in the presence of other cytokines, such as vascular endothelial growth factor (VEGF), Ang-2 stimulates an angiogenic response, whereas, in the absence of these cofactors, it elicits vessel regression [1]. Gene targeting studies have shown that Tie-1 and Tie-2 are essential for vascular development and maintenance. Studies in chimeric animals generated between normal embryonic cells and cells lacking Tie receptors indicated that these receptors are not required for differentiation and proliferation of definitive hematopoietic lineages in the embryo and fetus, but are specifically required during postnatal bone marrow hematopoiesis [4]. The interaction, at the level of stem cell niches, between quiescent hematopoietic stem cell cells (HSCs, expressing Tie-2) and the endosteal niche (producing Ang-1) induces the cellular adhesion of HSCs to osteoblastic cells, contribute to survival of HSCs and protect stem cells against various types of potentially dangerous cellular stresses [5], [6]. Furthermore, these studies have provided evidence that Ang-1 released by osteoblasts plays a critical role in inducing HSC quiescence [5]. Interestingly, when HSCs are induced to cycle, TIMP-3, a tissue inhibitor of metalloproteinase-3, inhibits Ang-1 signaling [7]. Ang-2, the other Tie-2 ligand, known to be an antagonist of Tie-2/Ang-1 signaling in angiogenesis, seems to act as an Ang-1 antagonist at the level of HSCs: in fact, while Ang-1 maintained long-term repopulating activity of HSCs, the addition of Ang-2 markedly interfered with the effects of Ang-1 [8].

In addition to its expression in the HSC/progenitor cell (HPC) compartment, Tie-2 is clearly expressed in the monocytic lineage [9]. Significant proportions of peripheral blood monocytes express Tie-2: these Tie-2^+^ monocytes are attracted in peritumoral areas through chemiotactic stimuli mediated via Tie-2 activation by Ang-1 triggering [10], [11]. These monocytes contribute to the process of tumor neoangiogenesis through paracrine mechanisms [10], [11]. Monocytic acute leukemia blast express elevated levels of Tie-2 on their membrane in association with the receptors of other endothelial growth factors [12].

Some observations suggest a possible role of the Angiopoietin/Tie-2 system in megakaryocytopoiesis. In fact, bone marrow immunohistochemical studies using an anti-Tie-2 monoclonal antibody have shown marked reactivity of megakaryocytes with this antibody [13]. On the other hand, it was provided evidence that Ang-1 is produced by human megakaryocytes under form of various isoforms exhibiting different biological properties [14]. Angiopoietins together with other angiopoietic factors, such as VEGF, FGF-2, PDGF and HGF, are stored in platelet alfa-granules: platelet-derived angiogenetic factors promote growth and proliferation of endothelial cells [15]. However, any possible role of angiopoietins in megakaryocytic differentiation/proliferation remains to be demonstrated. On the other hand, the Tie-2 induced signaling in megakaryocytic cells, as well as more generally in hematopoietic cells, remains to be explored.

To investigate the role of Ang-1/Ang-2 in the megakaryocytic compartment, we analyzed the expression and function of Ang-1, Ang-2 and Tie-2 on TPO-induced: a) UT7/mpl (UT7 cells engineered to express the TPO receptor, also known as c-mpl) [16], [17]; b) human HPCs purified from either cord blood (CB) or peripheral blood (PB). The experimental models are useful and complementary tools to investigate the Mk proliferation and differentiation processes. Indeed, when cultured in the presence of TPO, UT7/mpl, CB- and PB-HPCs proliferate and undergo Mk differentiation and maturation accompanied by nuclear polylobation, though at different extents in these various cellular systems. Thus, TPO-induced UT7/mpl cells show a high proliferative rate, but they only partially differentiate and polylobate [16], [17]. Instead, TPO-supplemented CB and PB HPCs reach terminal Mk differentiation [18]. However, CB cultures are characterized by a sustained Mk proliferation and limited polyploidization [19], [20], while in vitro grown PB-Mks proliferate less, but undergo a massive polylobation of the nuclei, underlying the occurrence of incoming polyploidization.

Our results indicate that Ang-1/Ang-2 may have a role in megakaryopoiesis. Particularly, Ang-2 is predominantly produced and released by immature normal megakaryocytic cells and by undifferentiated UT7/mpl cells and slightly stimulated TPO-induced cell proliferation. Ang-1 production is markedly induced during megakaryocytic differentiation/maturation and potentiated TPO-driven megakaryocytic differentiation. Blocking endogenously released angiopoietins partially inhibited megakaryocytic differentiation, particularly for that concerns the process of polyploidization.

**Figure 1:**
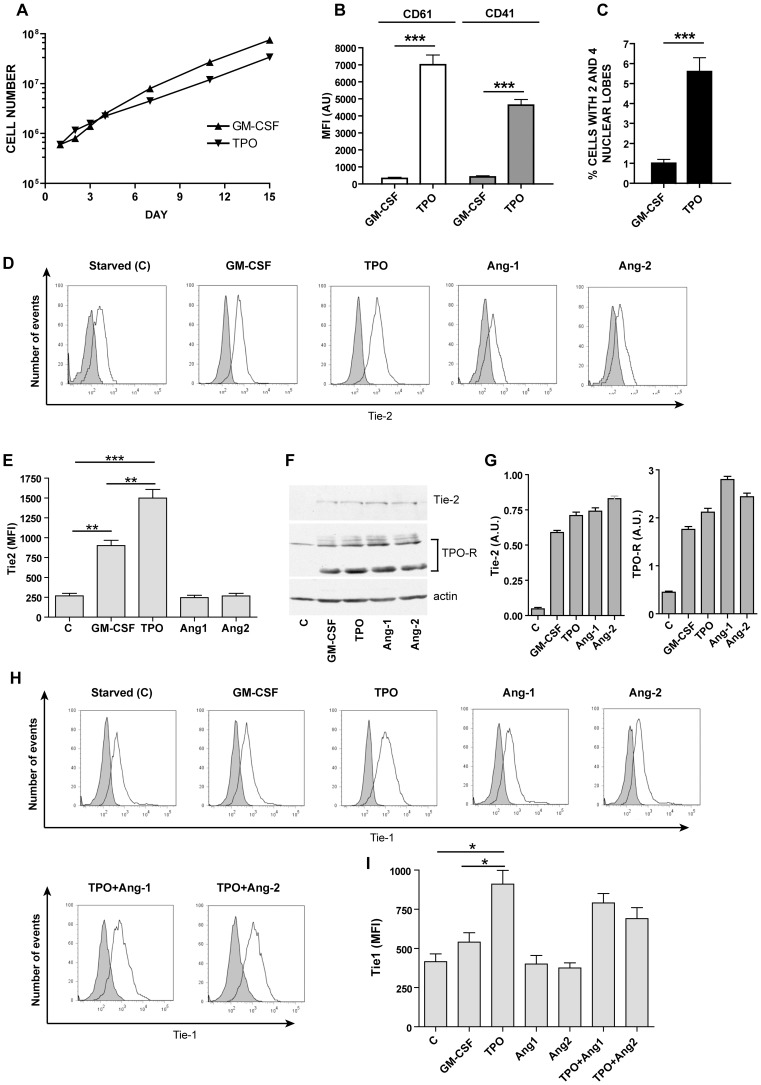
Growth, differentiation, Tie-2 and Tie-1 expression in UT7/mpl cells. A: Cell growth curve of UT7/mpl cells grown in the presence of GM-CSF or TPO. **B**: CD41 and CD61 (mean values ± SEM in three separate experiments) expression in UT7/mpl cells grown for 7 days either in the presence of GM-CSF or TPO. **C**: Proportion of cells with polylobated nuclei (mean values ± SEM in three separate experiments) at day 7 of either GM-CSF or TPO treatment. **D**: Flow cytometric analysis of Tie-2 expression in UT7/mpl cells grown either in the absence of growth factors (starved) or in the presence of either GM-CSF or TPO or Ang-1 or Ang-2 for 36 h. **E**: Mean Fluorescence Intensity (MFI) values of Tie-2 expression as evaluated by flow cytometry analysis. The data represent the mean values ± SEM observed in three separate experiments. **F**: Western blotting analysis of Tie-2 and TPO-R expression in UT7/mpl cells grown as above. **G**: Quantification of western blot results by band densitometry analysis (Mean Absorbance Units ± SEM from three separate experiments). **H**: Tie-1 expression in UT7/mpl cells. Flow cytometric analysis of Tie-1 expression in UT7/mpl cells deprived for 36 h of growth factors (starved) or grown for 36 h either in the presence of either GM-CSF, TPO, Ang-1, Ang-2, TPO+Ang-1 or TPO+Ang-2. In the first seven panels, from the top to the bottom a representative flow cytometry analysis of Tie-1 is shown. Mean Fluorescence Intensity (MFI) values (mean values ± SEM observed in three separate experiments) are reported in **I**. *, **, ***: p<0.05, p<0.01, p<0.001, respectively.

## Materials and Methods

### Ethics Statement

This study was specifically approved by the Institutional Review Board of the Istituto Superiore di Sanità and was in accordance with the principles of the Helsinki Declaration II. The written informed content was obtained from each patient.

### Cell Lines

GM-CSF-dependent human erythro-megakaryoblastic UT7 cell line [obtained from the European Collection Association of Cell Cultures, ECACC)] and UT7 expressing the full-length murine TPO receptor mpl (UT7-mpl) [15]–[16] were maintained in α-minimal essential medium supplemented with 10% fetal calf serum (FCS) and 10 ng/ml recombinant human GM-CSF. In some experiments UT7-mpl cells have been grown in the presence of 50 ng/ml human recombinant TPO.

In some experiments UT7-mpl cells, grown either with GM-CSF or TPO, were supplemented or not with 10 µg/ml Tie-2Fc (extracellular domain of Tie-2 fused to Fc region of human IgG, R&D Systems) and evaluated for cell growth, apoptosis and cell differentiation.

HUVEC cells (obtained from ECACC) have been grown in endothelial cell culture medium EGM2 (Lonza Company, Cologne, GmbH).

**Figure 2:**
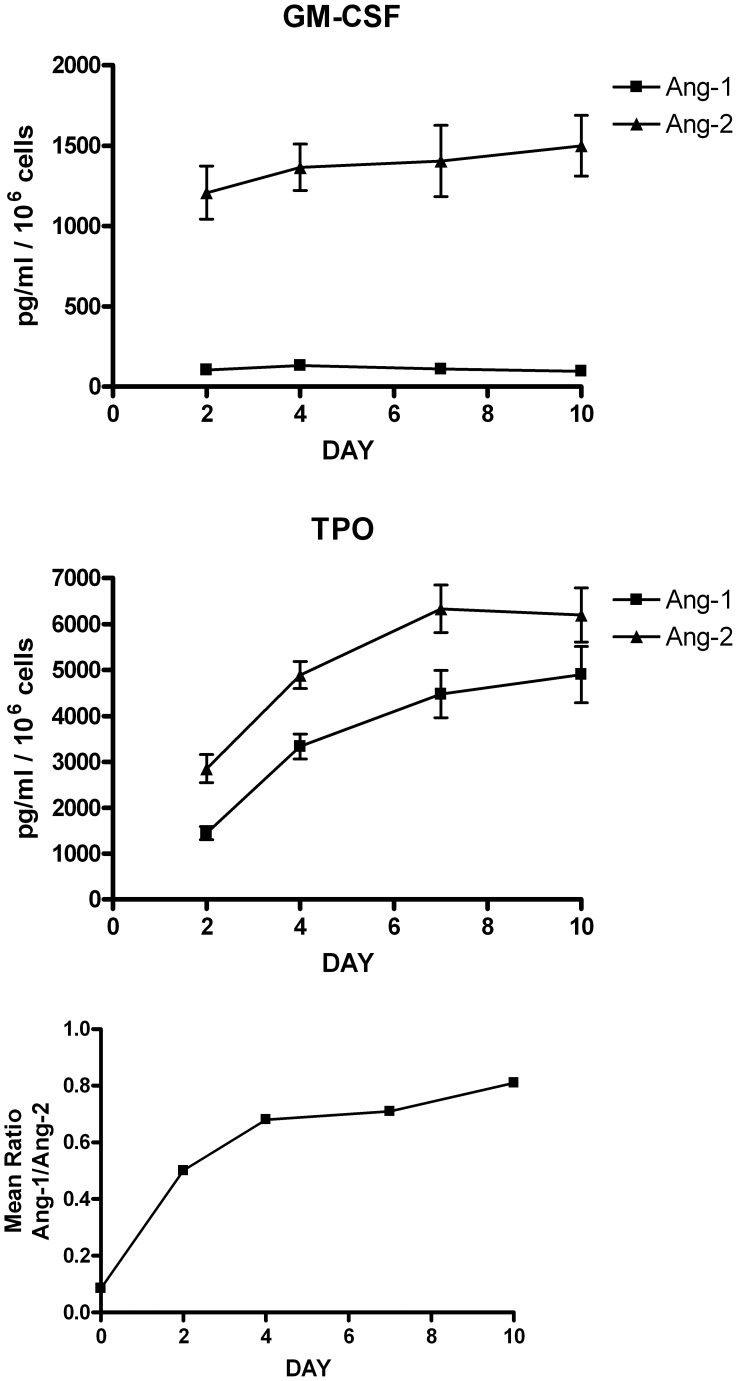
Release of angiopoietins during differentiation of UT7/mpl cells. UT7/mpl cells have been grown for 7 days in the presence of either GM-CSF (*Top Panels*) or TPO (*Middle Panels*) and the concentration of Ang-1 and Ang-2 in the culture supernatants was evaluated by a sensitive and specific ELISA assay. The levels of angiopoietins reported in the figure correspond to the accumulated amount of angiopoietins during the culture. Mean values ± SEM observed in three separate experiments. In the bottom panel the ratio Ang-1/Ang-2 at various days of culture is plotted.

### Peripheral Blood and Cord Blood Human Progenitor Cell (HPC) Purification and Culture

Peripheral blood (PB) mononuclear cells were obtained from buffy coats removed from blood donation of normal healthy subjects. Cord blood (CB) was obtained after informed consent from healthy full-term placentas according to institutional guidelines. Human CD34^+^ cells were purified from PB or CB by positive selection using the midi-MACS immunomagnetic separation system (Miltenyi Biotec, Bergisch Gladabach, Germany) according to the manufacturer’s instructions. The purity of CD34^+^ cells was assessed by flow cytometry using a monoclonal PE-conjugated anti-CD34 antibody and was routinely over 95% (range comprised between 92–98%).

CD34^+^ progenitors were cultured in serum-free medium in the presence of 100 ng/ml TPO to induce selective megakaryocytic cell differentiation (18). In some experiments, CD34^+^ progenitors were grown with TPO in combination with Tie-2/Fc, Ang-1 or Ang-2 (at the concentrations of 10 μg/ml, 100 ng/ml and 100 ng/ml, respectively). Serum-free medium was prepared as it follows: freshly prepared Iscove’s modified Dulbecco’s medium was supplemented with bovine serum albumin (BSA was treated with dextran/chorcal and deionized and added at a final concentration of 10 mg/ml), pure human transferrin was fully saturated with iron and added at a final concentration of 700 μg/ml, purified human low-density lipoprotein (40 μg/ml), insulin (dissolved in Hanks’ balanced salt solution and added at a final concentration of 10 μg/ml), sodium pyruvate (10^−4^ mol/L), L-glutamine (2×10^−3^ mol/L), rare inorganic elements [sodium selenite Na_2_SIO_3_ (5×10^−7^ M) MnSO_4_ (1×10^−9^ M), (NH_4_)_6_MO_7_O_24_ (1×10^−9^ M), NH_4_VO_3_ (5×10^−9^ M), NICl_2_ (5×10^−10^ M), SnCl_2_ (5×10^−10^ M) and FeSO_4_ (4×10^−8^ M)] supplemented with iron sulfate (4×10^−8^ mol/L) and nucleosides (10 μg/ml each). All these reagents were purchased from Sigma Co (St Louis, USA). In these culture conditions a cell progeny of cells 93±3% CD61^+^ is generated [18].

**Figure 3:**
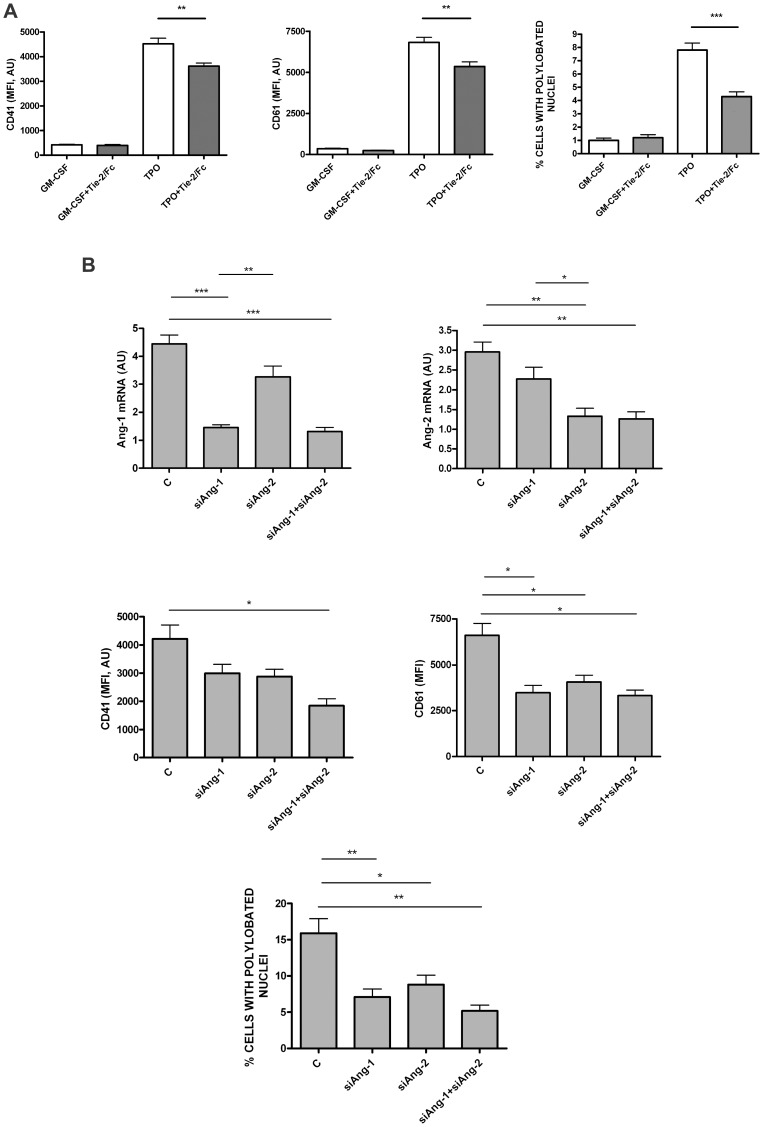
Effect of endogenous angiopoietin neutralization on Mk differentiation of UT7/mpl cells. **A-** Effect of endogenous angiopoietin neutralization by Tie-2/Fc on Mk polyploidization in UT7/mpl cells. UT7/mpl cells have been grown for 7 days either in the presence of GM-CSF or TPO and either in the absence or in the presence of Tie-2/Fc; after 7 days of culture the cells have been harvested and evaluated for CD41 and CD61 expression by flow cytometry (*top* and *middle panels*) and for the proportion of cells with polylobated nuclei (*bottom panel*). Mean values ± SEM observed in three separate experiments. ** p<0.01; *** p<0.001. **B-** Effect of siAng-1 RNA or siAng-2 RNA on Mk differentiation of UT7/mpl cells. UT7/mpl cells have been transfected with either a control scramble siRNA (C ) or siAng-1 RNA or siAng-2 RNA or siAng-2 RNA or both siAng-1+ siAng-2 RNAs and then grown for four days in the presence of TPO and assayed for Ang-1 mRNA (*left, top panel*) or Ang-2 mRNA (*right, top panel*), for Mk membrane CD41 (*left, middle panel*) or CD61 (*right, middle panel*) antigens and for the presence of polylobated nuclei (*bottom panel*). Mean values ± SEM observed in three independent experiments are shown. *, **, ***: p<0.05, p<0.01, p<0.001, respectively.

**Figure 4:**
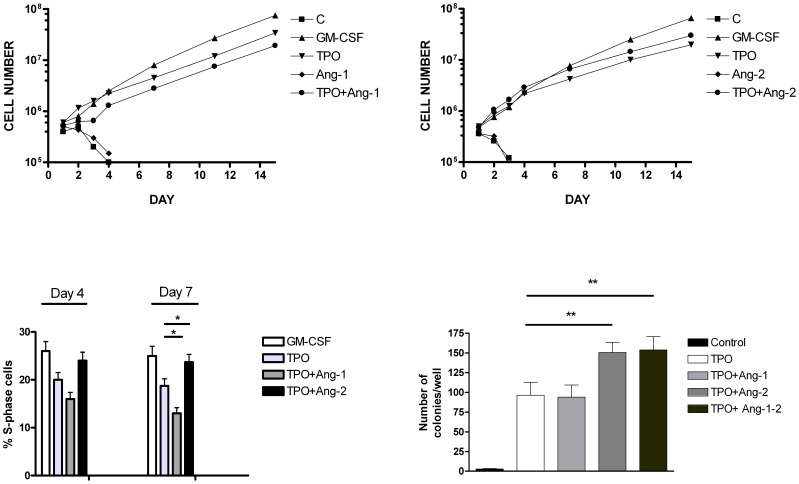
Effect of exogenous angiopoietins on the growth of UT7/mpl cells. *Top Panels*: UT7/mpl cells have been grown either in the absence (C ) or in the presence of either GM-CSF, TPO, Ang-1, Ang-2, TPO+Ang-1 or TPO+Ang-2 and the number of living cells was determined at each day of culture. *Bottom Panel*, left: At day 4 and 7 of culture cell aliquots were harvested and analyzed for cell cycle by PI labeling and flow cytometry. The proportion of S phase cells (mean values ± SEM observed in three separate experiments) is reported. * p<0.05 *Bottom panel*, right: UT7/mpl cells have been plated in methylcellulose either in the absence (control) or in the presence of either TPO, TPO+Ang-1, TPO+Ang-2 or TPO+Ang-1+Ang-2 and after 10 days of *in vitro* culture the number of colonies was evaluated under an inverted microscope. Mean values ± SEM observed in three independent experiments are shown. ** p<0.01.

Cells were cultured at 37°C in a 5% CO_2_/5%O_2_/90%N_2_ atmosphere. The differentiation stage of unilineage cultures was evaluated by May-Grunwald-Giemsa staining (Sigma-Aldrich, St. Louis, Mo, USA) and cytologic analysis.

### Cell Transfection

Transient transfections of UT7/mpl cells with small interfering (si)RNA were carried out using Lipofectamine 2000 (Invitrogen, Carlsbad, CA, USA). Two chemically synthesized SiRNAs (Silencer Select Pre-designed and Validated siRNA) to Ang-1 (S1356) and Ang-2 (S1361), and scrambled siRNAs were purchased from Ambion and transfected at 10 nM final concentration. The following day, the cells were treated with 50 ng/ml human recombinant TPO. After 72 hours, expression of Ang-1 and Ang-2 was determined by real-time PCR.

### Morphological Studies

UT7-mpl cells were collected every 2–3 days and concentrated on glass slides by cytocentrifugation. Cell morphology was examined after May-Grunwald-Giemsa staining. Morphological analysis was performed to assess both cell maturation and the number of nuclear lobes per cell.

**Figure 5:**
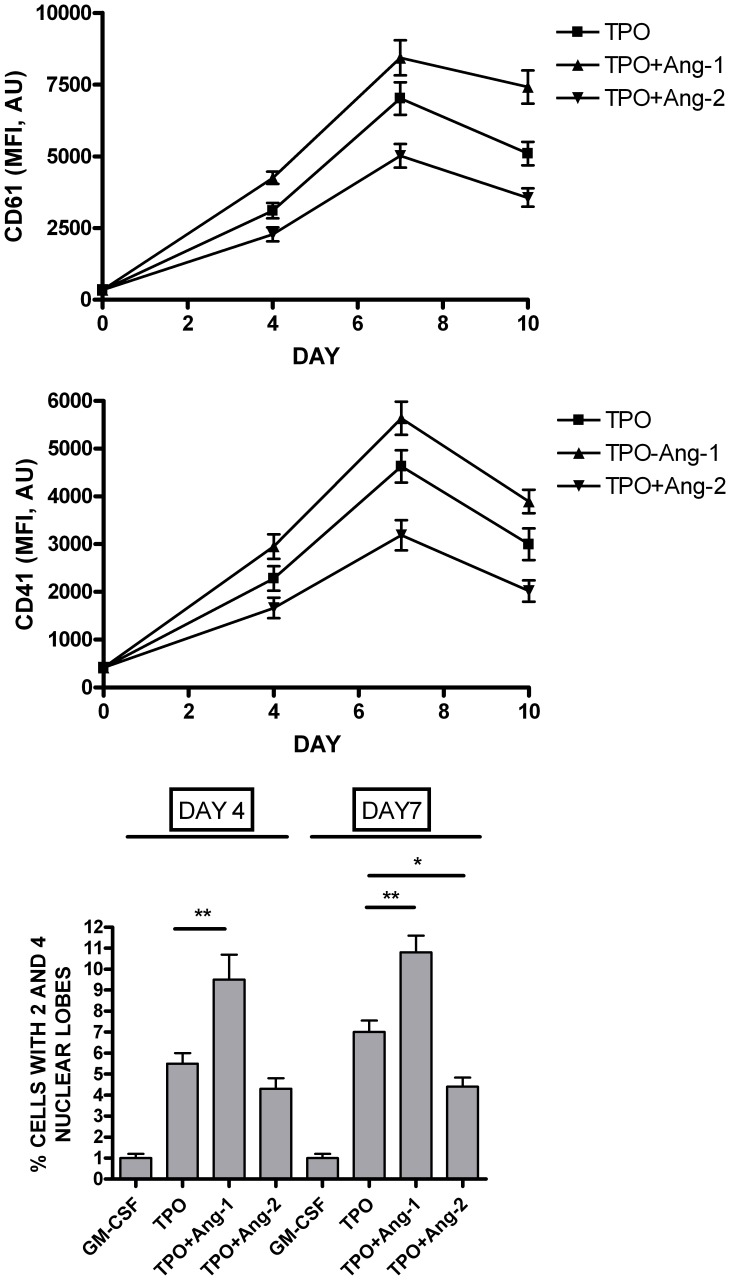
Effect of exogenous Ang-1 and Ang-2 on TPO-driven Mk differentiation of UT7/mpl cells. UT7/mpl cells have been grown in the presence of TPO alone or in combination with either Ang-1 or Ang-2 and at various days of culture the expression of CD61 (*Top Panel*) and CD41 (*Middle Panel*) antigens by flow cytometry and the percentage of polylobated (i.e., with 2 or 4 nuclear lobes) nuclei was evaluated by microscopy inspection of individual MGG-stained cells (*Bottom Panel*). The data represent mean values ± SEM observed in three separate experiments. In the top and middle panels the difference between TPO and TPO+Ang-1 or TPO+Ang-2 was significant (p<0.05). * p<0.05; ** p<0.01.

### Flow Cytometry Analysis of Cell Surface Antigens

Cells treated or not treated for various days with growth factors, were washed twice in PBS and incubated 30 min at 4°C with 1 mg/ml of human immunoglobulins to saturate Fc receptor. After two washes with PBS, mouse monoclonal antibodies directly conjugated with fluorochromes or appropriate negative control (isotype-matched mouse immunoglobulins) were added and incubated 60 min at 4°C. After two additional washes, cells were resuspended in 1% formaldehyde and analysed for fluorescence on a FACS SCAN flow cytometer (Becton Dickinson, Mountain View, CA, USA). The percentage of positive cells and the Mean Fluorescence Intensity (MFI), a measure of relative density of the antigen on the surfaces of stained cells, were determined for each sample.

**Figure 6:**
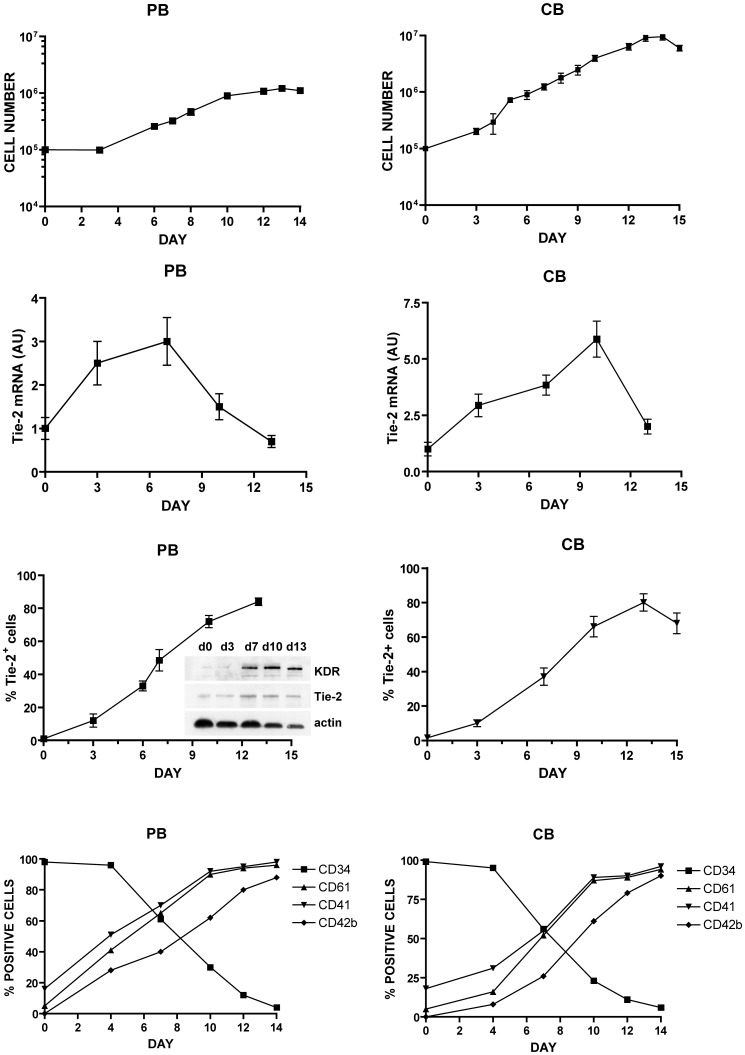
Tie-2 expression during Mk differentiation of cord blood or peripheral blood cord blood or peripheral blood CD34^+^ cells. Kinetics of Tie-2 expression during TPO-driven megakaryocytic differentiation of cord blood (CB, *Left Panels*) or cord blood (PB, *Right Panels*) CD34^+^ cells. Purified CB or PB CD34^+^ cells have been grown in serum-free medium in the presence of TPO and, at various days of culture, cell aliquots have been harvested and and analyzed for cell growth (*top panels*), for Tie-2 expression at mRNA and protein level (mean percentage of positive cells ± SEM observed in three separate experiments, *middle panels*) and for the expression of membrane differentiation markers CD34, CD41, CD61 and CD42b (*bottom panels*). In the *inset* the Tie-2 WB analysis on Mks grown from PB is reported. Mean values ± SEM observed in three separate experiments.

**Figure 7:**
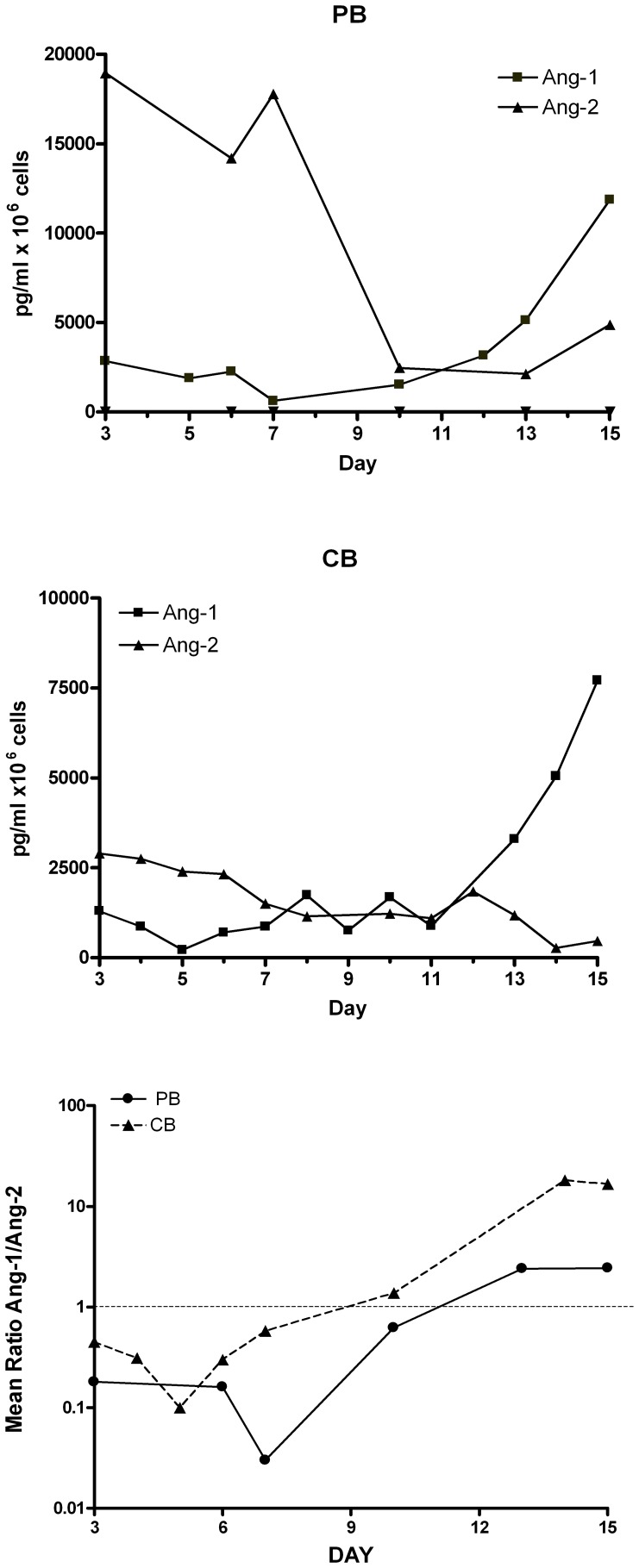
Release of angiopoietins by differentiating megakaryocytes. PB (*top panel*) or CB (*middle panel*) CD34^+^ cells have been grown in unilineage Mk cultures and culture supernatants have been harvested at various days of culture and evaluated for Ang-1 and Ang-2 content per 10^6^ cells. Mean values ± SEM observed in three separate experiments are shown. In the *bottom panel* the ratio Ang-1/Ang-2 at various days of culture is plotted.

### Cytokines for Cell Culture and Antibodies for Flow Cytometry

Human recombinant GM-CSF, TPO, Ang-1 and Ang-2 were obtained from R&D Systems (R&D Systems Inc., Minneapolis, USA).

Anti-CD9, -CD41, -CD42b and CD61 mAbs were obtained from Immunological Sciences, Rome, Italy. Anti-Tie2 mAb was purchased from R&D Systems. Anti-c-mpl mAb was obtained from Becton Dickinson, San Jose, CA, USA.

### Angiogenic Growth Factor Evaluation in Culture Supernatants

Ang-1 or Ang-2 concentration in cell supernatants at various days of culture was measured by using human specific enzyme-linked immunosorbent assays purchased from R&D Systems, according to manufacturer’s instructions. The limit detection of these assays was 10–20 pg/ml.

### Western Blot Analysis

To prepare total extracts, the cells were washed twice with cold phosphate-buffered saline and lysed on ice for 30 minutes with 1% Nonidet P40 lysis buffer (20 mM Tris-HCl pH 8.0, 137 mM NaCl, 10% glycerol, 2 mM EDTA) in the presence of 1 mM phenymethylsulfonyl fluoride, 1 mM dithiothreitol, 1 mM sodium orthovanadate, 2 µg/ml leupeptin, and 2 µg/ml aprotinin. Cell debris was removed by centrifugation at 10,000 rpm for 10 min at 4°C, and protein concentration of supernatants was determined by the Biorad protein assay (Richmond, CA, USA). Aliquots of cell extracts containing 30–50 µg of total protein were resolved by 7.5% SDS-PAGE under reducing and denaturing conditions and transferred to nitrocellulose filter. The blots were blocked using 5% non-fat dry milk in TBST (10 mM Tris-HCl pH 8.0, 150 mM NaCl, 0.1% Tween 20) for 1 h at room temperature, followed by incubation with primary antibodies. After washing with TBST, the filters were incubated with the appropriate horseradish-peroxidase-conjugated secondary antibodies (Bio-Rad) for 1 h at room temperature. Immunoreactivity was revealed using an ECL detection kit (Pierce, USA).

In WB experiments the following antibodies were used: Tie-1 (clone8C9) was from Novus Biologicals, Tie-2 (mAb 05584) from Upstate; TPO-R (mAb AP1016), p-Stat5A/B (mAb AF4190), p-p38 (mAb AF869) and p-ERK 1/2 (mAb AF1018), are purchased from R&D Systems, Minneapolis, USA; p-AKT (mAb 9271), p38 MAP kinase (mAb 9212), ERK 1/2 (mAb 4695), STAT5 (mAb 9363) and AKT (mAb 9272) are purchased from Cell Signaling, Beverly, MA, USA).

**Figure 8:**
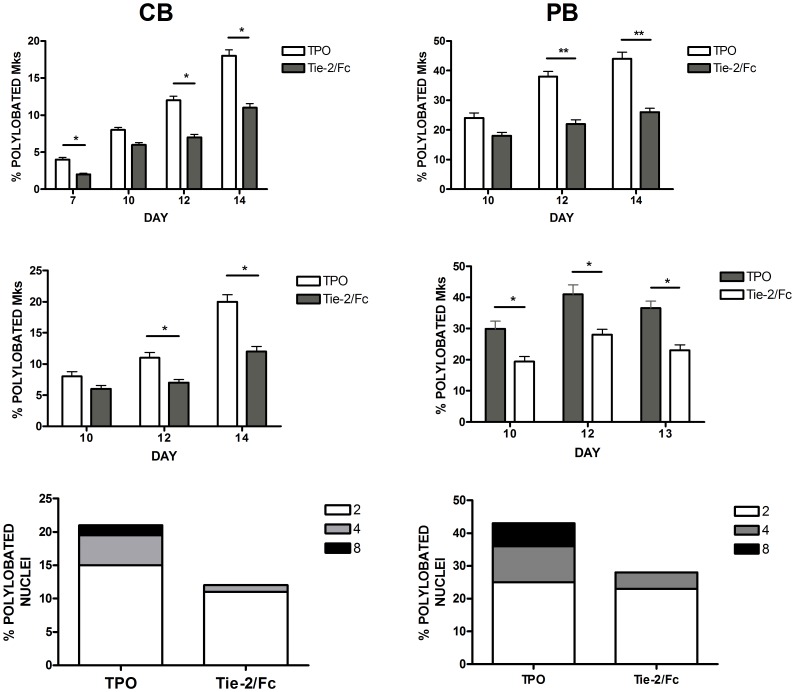
Effect of endogenous angiopoietin neutralization by Tie-2/Fc on Mk polyploidization in normal megakaryocytes. CB (*left panels*) or PB (*right panels*) CD34^+^ cells have been grown in the presence of TPO to induce Mk differentiation either in the absence (TPO ) or in the presence of Tie-2/Fc (TPO+Tie-2/Fc); Tie-2/Fc was added either at day 0 of culture (*top panels*) or at day 7 of culture (*middle and bottom panels*). The data reported in bottom panels are derived from day 14 CB Mks and day 12 PB Mks. Cell aliquots were harvested at various days of culture, cytocentrifuged, stained with MGG and through microscopic inspection the proportion of cells displaying 2, 4 or 8 nuclei lobi was determined. The data reported in the Figure represent the mean values ± SEM observed in three separate experiments. * p<0.05.

**Figure 9:**
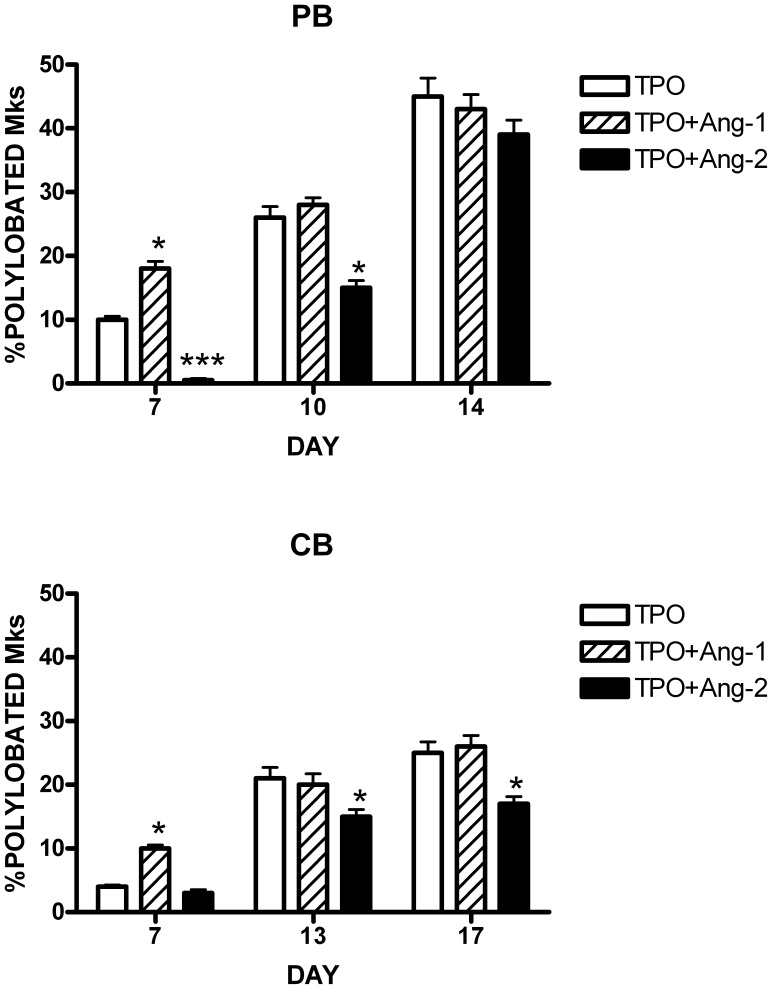
Effect of exogenous angiopoietins on TPO-driven Mk differentiation of HPCs. CD34^+^ HPCs purified either from CB (*top panel*) or PB (*bottom panel*) have been triggered to Mk differentiation with TPO either in the absence of growth factors (TPO ) or in the presence of either 100 ng/ml Ang-1 (TPO+Ang-1) or 100 ng/ml Ang-2 (TPO+Ang-2) and then assayed for Mk ploidy by evaluating the percentage of polylobated Mks. The data reported in the Figure represent mean values ± SEM observed in three separate experiments. * p = <0.05 when compared to the corresponding TPO-treated sample. *** p = <0.001 when compared to the corresponding TPO-treated sample.

### Colony Assay

UT7-mpl cells (500 cells/ml/dish) have been plated in methylcellulose semisolid medium supplemented with various growth factors (GM-CSF or TPO or Ang-1 or Ang-2 or TPO+Ang-1 or TPO+Ang-2). After 10–12 days of culture the number of colonies was evaluated by inspection under an inverted microscope.

### RNA Analysis

Total RNA was extracted by guanidinium isothiocyanate/CsCl method and reverse-transcribed using random primers-RT kit (Invitrogen), according to the manufacturer’s procedure.

Quantitative real-time (qRT)–PCR analysis was performed by TaqMan technology, using the ABI PRISM 7700 DNA Sequence Detection System (Applied Biosystems, Foster City, CA,USA). Commercial ready-to-use primers/probe mixes were used (Assays on Demand Products, Applied Biosystems) for GAPDH, Tie-2, Ang-1, Ang-2 and KDR.

### Statistical Analysis

Statistical significance of differences observed between different experimental groups was determined using a Student’s *t* test. The minimal level of significance was a *P* value below 0.05.

**Figure 10:**
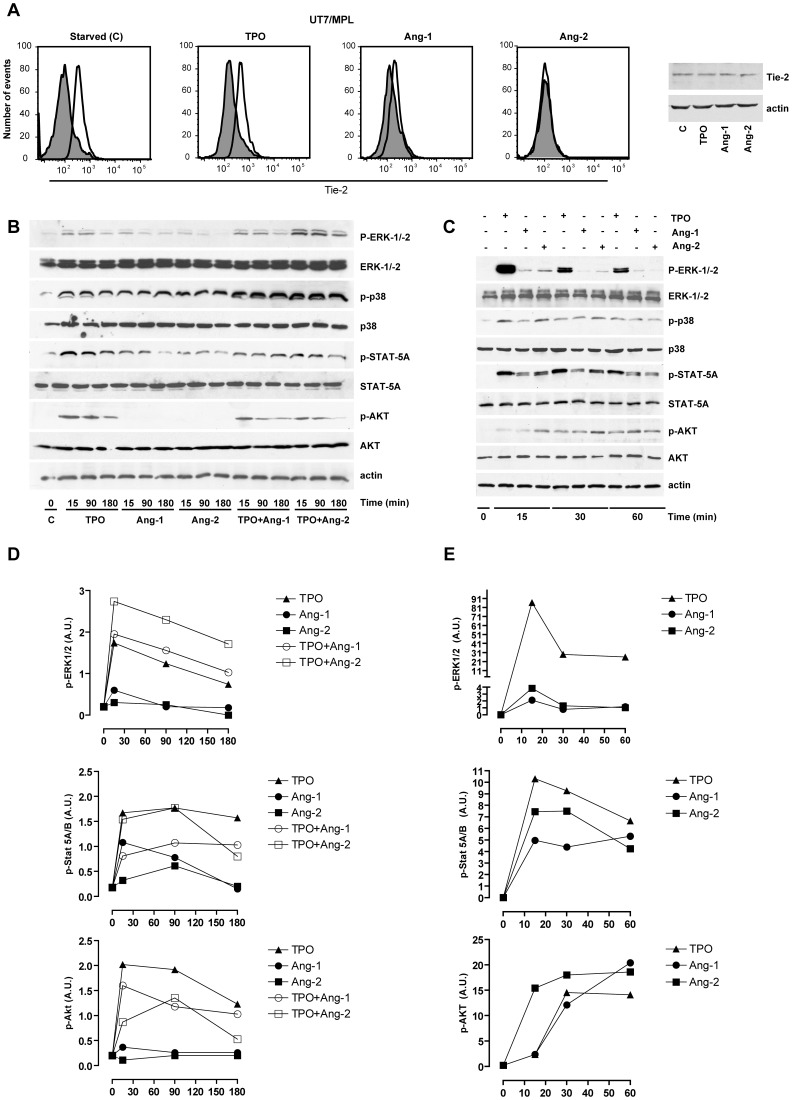
Angiopoietin and TPO-induced cell signaling in UT7/mpl cells and in normal megakaryocytes. *A –* Flow cytometry and Western Blot analysis of Tie-2 expression in UT7/mpl cells starved for 16 h of growth factors and then stimulated for 15 min either in the absence (C ) or in the presence of either TPO or Ang-1 or Ang-2. *B* – UT7/mpl cells have been starved for 16 h of growth factors and then stimulated for various times (15, 90 and 180 min) with either TPO or Ang-1 or Ang-2 or TPO+Ang-1 or TPO+Ang-2. The cells were then harvested, lysed and analyzed by western blotting for the expression of p-ERK-1/−2, p-38, p-STAT-5A and p-AKT and their non-phosphorylated forms. Beta-actin was used to normalize for sample loading. A representative experiment out of three performed is shown. *C* – Day 7 megakaryocytes derived from unilineage cell cultures of normal CB CD34^+^ cells have been first starved for 6 h of exogenous growth factors and then stimulated for various times (15, 30 or 60 min) with either TPO or Ang-1 or Ang-2. Cells were then washed, lysed and processed as above. Beta-actin was used to normalize for sample loading. *D-E* Densitometric analysis of WB autoradiograms, performed on UT7/mpl (*panel D*) or d7 PB MKs (*panel E*) expressed as absorbance arbitrary units. The results represent the mean values observed in three separate experiments.

**Figure 11:**
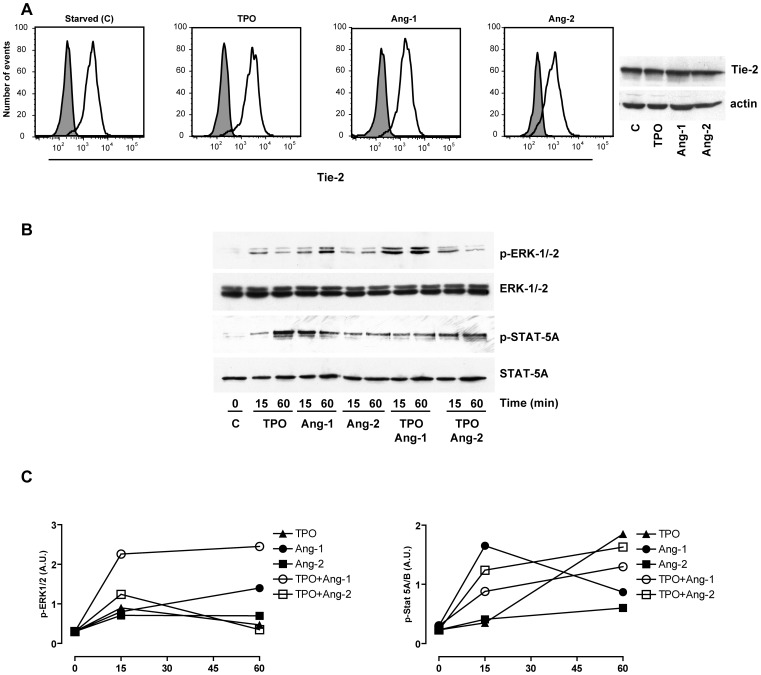
Angiopoietin and TPO-induced cell signaling in HUVEC cells. HUVEC cells, grown under standard endothelial cell culture conditions, have been starved overnight of growth factors and stimulated as indicated in panel B of Fig.10. *A –* Flow cytometry and Western Blot analysis of Tie-2 expression in HUVEC cells starved of growth factors and then stimulated for 15 min either in the absence (C ) or in the presence of either TPO or Ang-1 or Ang-2. *B and C* - WB and densitometric quantification (*top* and *bottom* panels, respectively).

## Results

### Tie-2 Expression and Angs Release in TPO-supplemented UT7/mpl Cells

To investigate the possible role played by Tie-2 and its ligands Ang-1 and Ang-2 in megakaryocitic growth and differentiation, we used the UT7/mpl cell line (GM-CSF dependent human megakaryoblastic UT7 cell line, expressing the full-length murine mpl) able to grow either in the presence of GM-CSF or in TPO-supplemented medium [15], [16]. In this cell line, TPO induces growth at slightly lower levels than GM-CSF (Fig. 1, A). In concomitance with the proliferative induction, TPO promotes also MK differentiation, as shown by the increase of expression of the early MK markers CD61 and CD41 (Fig.1, B) and the induction of polylobated nuclei (Fig.1, C). Nevertheless, TPO was unable to induce the expression of late MK membrane markers such as CD9 and CD42b (data not shown). Both GM-CSF and TPO induce Tie-2 expression as detected by flow cytometry (Fig.1, D, E) and WB analysis (Fig.1, F, G) and, whereas starved cells (cells deprived for 36 h of growth factors) express it at low levels. Interestingly, Tie-2 expression was higher in TPO-stimulated than in GM-CSF-grown UT7/mpl cells (see the results of MFI analysis, Fig.1, E). Cells grown for 36 h in the presence of 25 ng/ml Ang-1 or Ang-2 displayed Tie-2 expression similar to GM-CSF-grown cells by WB analysis, whereas flow cytometry analysis showed decreased plasma membrane Tie-2 expression, probably due to ligand-induced receptor internalization (Fig.1, D–G).

As expected, UT7/mpl cells display high levels of mpl (Fig. 1, F, G).

Since previous studies provided evidence that Tie-1 was expressed in erythroleukemic cell lines [21] and in human megakaryocytes [22] it seemed of interest to evaluate the effect of TPO on Tie-1 expression in UT7/mpl cells. Cell labeling experiments using a monoclonal antibody specifically interacting with the ectodomain of Tie-1 were carried out. These experiments showed that: (i) Tie-1 expression was equally detectable both in UT7/mpl cells deprived of growth factor or grown in the presence of GM-CSF; (ii) incubation of UT7/mpl cells in the presence of either Ang-1 or Ang-2 induces a slight, but not significant decrease of Tie-1 expression; (iii) TPO elicited a significant (p<0.05) increase of Tie-1 expression over the values observed for cells grown in the presence of GM-CSF (Fig. 1 H, I).

Then, we evaluated Angs release into the extracellular medium in UT7/mpl cell line. In GM-CSF growing UT7/mpl cells, Angs are released at a constant rate during culture. In particular, Ang-2 release is about 1.2–1,4 ng/ml/10^6^ cells whereas lower amounts of Ang-1 (about 0.1 ng/ml) are secreted (Fig.2, top panel). UT7/mpl cells induced by TPO markedly and progressively increased the release of angiopoietins and, particularly, of Ang-1 release: after ten days of TPO-treatment Ang-1 and Ang-2 were 4901±615 and 6199±588 pg/m, respectively) (Fig.2, middle panel). Interestingly, TPO-treated UT7/mpl showed an increase of Ang1/Ang-2 ratio up to 10 fold with respect to cells grown in the presence of GM-CSF (Fig.2, bottom panel).

### Sequestration of Angiopoietins: Effect on Proliferation and Differentiation of UT7/mpl Cell

The observation that in UT7/mpl megakaryoblasts, TPO induces a higher Angs release than GM-CSF treatment, led us to investigate a possible autocrine role of Angs on UT7/mpl growth and differentiation. Thus, we have treated TPO-supplemented UT7/mpl with Tie-2/Fc, a soluble receptor capable to bind and to neutralize the angiopoietins released in culture. Tie-2/Fc treatment on TPO-supplemented cells induced a slight inhibition of cell growth at culture day 2, while no difference was observed at later times (data not shown). Furthermore, Tie2/Fc slightly reduced the expression of CD41 and CD61 (Fig.3 A, *top* and *middle panels*). The effect of angiopoietin blockage was more evident at the level of MK ploidy: in fact, Tie-2/Fc addition reduced of about 50% the proportion of TPO-induced UT7-mpl cells displaying polylobated nuclei (Fig. 3 A, *bottom panel*).

The experiments carried out with Tie-2/Fc did not allow to distinguish between the potential effects selectively related to Ang-1 from those dependent from Ang-2. To verify this point experiments using specific si-RNA for Ang-1 or si-RNA for Ang-2 have been carried out. These experiments provided evidence that in TPO-treated UT7/mpl cells both Ang-1 siRNA and Ang-2 siRNA elicited a significant decrease of the expression of Mk membrane marker CD61 and of the proportion of polyploid cells (Fig. 3B); in contrast, a significant decrease of CD41 expression was induced only by the combined addition of both siRNAs (Fig. 3B).

### Angiopoietins Addition: Effect on TPO-induced Growth and Differentiation of UT7/mpl Cells

We then explored the effect of exogenous Ang-1 or Ang-2, added alone or in combination with TPO, on the growth of UT7/mpl cells. GM-CSF-supplemented cells were used as a growth positive control. Ang-1 or Ang-2 alone were unable to sustain the growth and to maintain the survival of UT7/mpl cells, whereas they could affect the growth of the cells in combination with TPO. In particular, Ang-1 moderately reduced (% of reduction 32±5.2) while Ang-2 slightly increased the growth of TPO-treated UT7/mpl cells (% of increase 38±6.4) (Fig.4, *top panels*). In line with these findings, the analysis of cell cycle showed that: TPO-treated cells displayed a moderate decrease of the percentage of S-phase cells compared to GM-CSF-treated cells (p<0.05); a further decrease was evident if Ang-1 was added to TPO-treated cells, particularly at day 7 of culture (p<0.05); finally, cells grown in the presence of TPO+Ang-2 showed a slight, but significant (p<0.05) increase of S-phase cells percentage compared to cells grown in the presence of TPO alone (Fig.4, *bottom panels*). Moreover, we observed that in UT7/mpl cells, TPO was able to support colony formation, whose number was potentiated by Ang-2 (p<0.01), but not by Ang-1 (Fig.4, *bottom panels*).

To explore whether Ang-1 and Ang-2 could modulate TPO-induced MK differentiation and polyploidyzation, we analyzed MK membrane antigens expression and cellular polylobation. The experiments carried out on UT7/mpl by adding Angs showed that Ang-1 potentiated, while Ang-2 decreased TPO-induced CD41 and CD61 expression (Fig.5). The opposing effects of the Angs were also observed by the cellular ploidy analysis: Ang-1 increased, while Ang-2 reduced the percentage of UT7/mpl polylobated cells (Fig.5).

### Tie-2 Expression and Angiopoietins Release in TPO-stimulated Human HPCs

Analyses of Angs and Tie-2 expression and modulation in UT7/mpl cells suggest that Ang/Tie-2 loop modulates MK proliferation and differentiation. However, leukemic UT7/mpl cells never reach complete differentiation with acquisition of polylobated nuclei. Thus, to implement our studies on MK differentiation, we used CB (*left panels*) and PB (*right panels*) purified HPCs, cultured in TPO-supplemented serum-free liquid media. In these experimental conditions, both CB and PB HPCs are triggered to proliferate and differentiate along the MK lineage, giving rise to a pure and fully differentiated MK progeny (Fig 6 *left and right panels*, respectively). Notably, CB-HPCs show a higher proliferative rate than PB-HPCs (Fig.6, *top panels*); both CB and PB MK cells express early and late MK markers (Fig.6, *bottom* panels). On the other hand, mature PB-MKs (44.5±4.8%) show more polylobated nuclei than mature CB-MKs (20.5±2.8%). To investigate whether proliferative and differentiative changes were related to Tie-2/Angs, we analysed Tie-2 expression in MK unilineage cultures. Tie-2 was evaluated both at protein level (Western blot and flow cytometry) and at mRNA level (real-time PCR). Flow cytometry and WB experiments showed a progressive increase of Tie-2 positive cell percentage at various days of culture, peaking at day 10–13, when megakaryocytic cells reached a full maturation (Fig.6, *middle panels* and *inset*). Tie-2 mRNA expression increased in both PB and CB MKs during the first days of culture, reaching a peak at day 7 and 10, respectively. At later days of culture Tie-2 mRNA expression declined (Fig. 6, *middle panels*).

Tie-2-modulated expression during megakaryopoiesis encouraged us to explore also the possible changes of angiopoietin release in the extracellular medium in TPO-induced HPCs from both PB and CB (Fig. 7).

In TPO-induced HPCs, Ang-2 is predominantly produced during early stages of MK differentiation (i.e., from day 3 to day 7 of culture), while in maturing MK cells (i.e., from day 7 to day 10) Ang-1 progressively accumulates, until it exceeds Ang-2 in fully mature MK (i.e. from day 11 to the end of culture) (Fig.7, *top* and *middle panels*). As a consequence of these changes, the Ang-1/Ang-2 ratio reflects a sharp inversion during MK differentiation (Fig. 7, *bottom panel*), showing an initial decline of Ang-1/Ang-2 ratio from day 2–3 to day 7 of culture, followed by a marked and progressive increase of the Ang-1/Ang-2 ratio during later phases of Mk differentiation (i.e., from day 7 to day 14–15), suggesting the existence of a functional switch in the type of angiopoietin produced during Mk differentiation. It is of interest to note that early PB megakaryocytes release higher levels of Ang-2 than early CB megakaryocytes, while mature CB Mks release more Ang-1 than mature PB Mks (Fig. 7).

### Sequestration of Angiopoietins: Effect on MK Cell Proliferation and Differentiation of TPO-triggered HPCs

As above mentioned the analysis of Ang-1/Ang-2 ratio in normal MK cultures shows the occurrence of two distinct phases: I) Ang1−/Ang-2 decreasing phase (early MK cultures); II) Ang-1/Ang-2 increasing phase (late Mk cultures from day 5 in CB and from day 7 in PB-MK cells) (Fig.7). The UT7/mpl cell line triggered by TPO exclusively shows an increasing Ang-1/Ang-2 ratio (Fig. 2). These observations suggest that UT7/mpl cells may behave similarly to normal MK cultures during the Ang-1/Ang-2 increasing phase, corresponding to day 7–15 of culture.

Thus, we have cultured normal MK cells in the presence of the soluble receptor Tie2-Fc, starting from either culture day 0 or day 7. In these experiments, independently of timing of Tie-2/Fc addition, Ang sequestration resulted in a pronounced inhibitory effect on MK ploidy (Fig.8, *top* and *middle panels*), as shown by a marked reduction of the proportion of MKs displaying 2, 4 or 8 nuclear lobes (Fig.8, *bottom panels*). Importantly, the inhibitory effect of Tie-2/Fc was observed in both CB and PB MK cultures.

### Angiopoietin Addition: Effect on Mk Differentiation of TPO-triggered HPCs

Given the sequential production of angiopoietins during normal Mk differentiation/maturation it seemed of interest to analyze the effect of exogenous Ang-1 or Ang-2 addition on Mk polyploidization. To this end, CD34^+^ HPCs purified either from CB or PB have been grown in the presence of TPO alone or TPO+Ang-1 or TPO+Ang-2. Ang-1 addition resulted in an accelerated kinetics of Mk polyploidization, as shown by the observation that at day 7 TPO+Ang-1 supplementation resulted in significantly higher proportion of polyploid Mks both in CB and CB Mk cultures (Fig. 9). In contrast, Ang-2 addition elicited an opposite effect, inducing a reduced Mk polyploidization, particularly evident at early culture time points (Fig. 9). These observations suggest that the sequential angiopoietin release observed during Mk differentiation may have important implications for the control of Mk maturation.

### Angiopoietin-driven Signaling in UT7/mpl and Human MKs

In order to evaluate the effect of angiopoietins on cell signaling, UT7/mpl cells have been starved of growth factors overnight (16 h) and then exposed for 15, 90 or 180 minutes in the presence of either TPO, or Ang-1, or Ang-2, or TPO+Ang-1 or TPO+Ang-2. Notably, after overnight starvation Tie-2 receptor was still clearly expressed on plasma membrane, as shown by flow cytometry analysis (Tie-2 MFI corresponding to 332±47 for starved cells) (Fig. 10A). A short exposure (15 min) of UT7/Mpl cells to Ang-1 or Ang-2 elicited a rapid and pronounced downmodulation of Tie-2 (see flow cytometry analysis reported on the left panels of Fig. 10A), seemingly due to receptor internalization since total Tie-2 levels, as detected by Western Blot analysis, remained unchanged (see WB analysis reported on the right panel of Fig. 10A). TPO induced ERK, AKT and Stat5 activation, while the two angiopoietins were able to induce a weak ERK and Stat5 activation, but were unable to activate AKT (Fig. 10B and D). These observations were well in line with the proliferation and survival studies, showing the TPO, but not Ang-1 or Ang-2, were able to support the survival and proliferation of UT7/mpl cells. Interestingly, Ang-2, but not Ang-1, added concomitantly with TPO induced an increase of TPO-driven p-ERK-1/−2 induction, in line with the capacity of Ang-2, but not Ang-1, to potentiate the TPO-driven proliferation (Fig.4). The contemporaneous addition of Ang-1 or Ang-2 with TPO did not modify the kinetics at the level of TPO-induced Stat-5 and AKT activation (Fig. 10B and D).

The experiments carried out in PB-megakaryocytes at culture day7 (Fig.10C and E) basically confirmed those obtained in UT7/mpl cells (weak activation of ERK-1/−2 and Stat-5 by Ang-1 or Ang-2), with the exception of AKT, whose phosphorylation is induced in megakaryocytes by Ang-1 or Ang-2 (Fig. 10C and E), at variance with UT7/mpl cells, where angiopoietins were unable to induce AKT phosphorylation.

Finally, a last set of experiments was carried out on HUVEC cells to compare the angiopoietin signaling observed in hematopoietic cells with the “canonical” signaling induced by these angiogenetic growth factors in endothelial cells. Previous studies have shown that HUVEC cells express c-mpl and respond to TPO in terms of activation of cell signaling pathways, of endothelial cell motility and neoangiogenesis [23], [24]. These cells express higher Tie-2 levels than UT7/Mpl cells: a short exposure of these cells to Ang-1 or Ang-2 elicited a slight decrease of membrane Tie-2 expression (see flow cytometry analysis on the left panels of Fig. 11A), but not of total Tie-2, as detected by Western Blot analysis (see WB analysis on the right panels of Fig. 11A). These experiments showed that both angiopoietins, as well as TPO, were potent activators of p-ERK-1/−2 and Stat-5 in HUVEC cells (Fig. 11B and C). It is of interest to note that Ang-1 was a more potent p-ERK-1/−2 and Stat-5 activator than Ang-2 (Fig. 11B and C). Furthermore, Ang-1, but not Ang-2, potentiated the stimulatory effect of TPO on p-ERK activation (Fig.11B and C).

## Discussion

Although VEGF is the most important angiogenic mediator from a physiologic point of view, other factors, such as angiopoietins, crosstalk and cooperate with it in the modulation of angiogenesis, vascular repair and remodeling [1], [25]. VEGF production in megakaryocytic cells is promoted by TPO [26], [27] and plays a role in megakaryocytic differentiation and maturation promoted by TPO [28], [29]. However, few data are available on the expression and function of angiopoietins/Tie-2 in megakaryocytic cells [13], [14]. Therefore, it seemed of interest to evaluate the possible role of angiopoietin/Tie-2 in megakaryocytic proliferation/differentiation and the signaling triggered by Angs in hematopoietic cells. Our results based on the analysis of normal HPCs triggered to megakaryocytic differentiation and of UT7-mpl cells induced to megakaryocytic differentiation show for the first time that during Mk differentiation the Tie-2 receptor is increasingly expressed on the cell membrane, that angiopoietins are produced and released in the extracellular milieu and that Angs blockade interferes with Mk proliferation, differentiation and polyploidization, thus suggesting the existence of a possible autocrine role for Ang-1/Ang-2 in megakaryocytic proliferation and differentiation. Particularly, our results indicate that at early stages of megakaryocytic differentiation Ang-2 is predominantly produced and stimulates TPO-driven proliferation; on the other hand, Ang-1 is predominantly produced by differentiating megakaryocytic cells and inhibits TPO-driven proliferation and improves megakaryocytic differentiation. These results, together with previous observations suggest that angiogenic growth factors released by differentiating megakaryocytic cells act as regulators of TPO-driven megakaryocytic differentiation/maturation.

Ang-2 has long been regarded as an antagonist for Ang-1, although more recent studies have indicated that Ang-2 may act as a context-dependent Tie-2 agonist, depending of the Ang-1/Ang-2 ratio [1], [30].

Previous studies have shown that megakaryocytes/platelets play an important role in the control of angiogenesis through the release of angiogenesis stimulators (such as VEGF, angiopoietins and basic fibroblast growth factor) and angiogenesis inhibitors (such as endostatin) contained in distinct populations of alpha-granules present in megakaryocytes and platelets [31]. Angiopoietins are released by platelets following thrombin stimulation and contribute to platelet-mediated control on angiogenesis and vascular stability [32]. In addition to the paracrine role in the control of the angiogenetic response, we suggest that the angiogenetic factors produced by megakaryocytes play an important role in the control of megakaryocyte differentiation/maturation acting through an autocrine/paracrine mechanism.

It is of interest to note that the UT7/mpl model was recently explored to define a possible link between megakaryocytic differentiation of these cells and VEGF binding activity [33]. These studies have shown that undifferentiated UT7/mpl cells expressed a functional VEGFR-2, leading to VEGF binding and VEGF-induced proliferation and apoptosis inhibition [33]. The megakaryocytic differentiation of UT7/mpl cells induced by phorbol myristate acetate was accompanied by a pronounced up-regulation of VEGF binding mediated by an increase of VEGFR-2 and Neuropilin-1 (a co-receptor of VEGFR-2) expression [33]. VEGFR-2 and Neuropilin-1 form a receptor complex that mediates cellular functions induced by VEGF in megakaryocytic cells [33]. This study and the present observations together suggest an important role of pro-angiogenic growth factors in megakaryocyte differentiation and function.

Another interesting finding of our study consists in the observation that angiopoietin production switches from Ang-2 to Ang-1 production during megakaryocytic differentiation. At the best of our knowledge this is the first report describing a switch in the type of angiopoietin production associated to cell differentiation. In this context, it is of interest to note that recent studies have shown that angiopoietin production is a general property of mature hemopoietic cells, angiopoietin production being predominant in neutrophils [34] and platelets [35], while mature monocytes are capable of releasing both Ang-1 and Ang-2 [36]. On the other hand, endothelial cells contain primarily Ang-2 [36].

Another interesting observation of our study is that the angiopoietin production by maturing megakaryocytes has an autocrine role as it affects the TPO-driven megakaryocyte proliferation and differentiation. In this context, the experiments of exogenous angiopoietin addition to HPCs triggered to Mk differentiation by TPO addition suggest a prominent role of the late-occurring Ang-1 release in promoting Mk maturation, while Ang-2 delays Mk maturation and seemingly favors Mk proliferation. This finding, supporting a role for endogenous angiopoietins in the control of cell proliferation, is in line with previous observations raised in other cellular systems [37]–[38]. Thus, it was shown that autocrine Ang-1 signaling in quiescent muscle stellate cells promote cell self-renewal [37], and autocrine Ang-2 production by endothelial cells acts as an autocrine protective factor in these cells [38].

We observed also that endogenously released angiopoietins may contribute, acting together with TPO, to the induction of megakaryocytic differentiation. Blocking endogenously released angiopoietins partially inhibited differentiation, particularly for that concerns the process of polyploidization. Few previous studies have supported a role for angiopoietins in the control of cell differentiation. In this context, some reports have indicated a possible positive role for Ang-1 in the neuronal differentiation of neural stem/progenitor cells [39].

It is important to note that the experiments of silencing of endogenous Angs have generated results in part at variance with those obtained through addition of exogenous Angs. However, this discrepancy can be explained taking into account previous studies carried out on endothelial cells [39]. In fact, these studies have shown that Ang-2 functions as a Tie-2 agonist when Ang-1 is absent or is present in a concentration up to 5 times higher than Ang-1; in contrast, when the Ang-2/Ang-1 ratio was >5, and particularly >20, Ang-2 resulted in an inhibitory effect on Ang-1-mediated Tie-2 activation [38]. In TPO-induced UT7/Mpl cells we observed an Ang-2/Ang-1 ratio of about 1.5 and, therefore, in this condition we expect that the endogenous Ang-1 and Ang-2 cooperate in activating Tie-2. In contrast, when we added exogenous Ang-2 we get a Ang-2/Ang-1 ratio >20, resulting in an inhibition of the Tie-2 activation elicited by endogenous Ang-1.

In a previous study we described the signaling induced by TPO during Mk differentiation [40]. Here, we provide evidence that angiopoietins are able to activate their membrane Tie-2 receptors and to induce a cell signaling in hematopoietic cells. Particularly, the experiments carried out in both UT7/mpl cells and in normal megakaryocytes show that angiopoietins are able to induce ERK1−/−2 and Stat-5 activation and through this mechanism seemingly cooperate with TPO in controlling megakaryocytic proliferation and differentiation. Thus, in UT7/mpl cells Ang-2 was able to potentiate TPO-driven ERK-1/−2 activation and through this mechanism may cooperate with TPO in sustaining megakaryocytic proliferation. However, it must be noted that the stimulation of ERK-1/−2 phosphorylation induced by angiopoietins alone in hematopoietic cells (either UT7/mpl or normal megakaryocytes) was weak, while both Ang-1 and Ang-2 induced a marked activation of ERK-1/−2 in endothelial cells [38], [41]. These differences in the level of ERK1−/−2 activation achieved by angiopoietins in hematopoietic and endothelial cells reflect the different capacity of these growth factors when act alone to support the growth of endothelial, but not of hematopoietic cells.

It is well known that angiopoietins may be bound at the cell surface not only by Tie-2, but also by Tie-1. Recent studies have shown that Tie-1 is able to form a complex at the cell surface with Tie-2: it is unclear whether the formation of this complex requires angiopoietin stimulation [42] or occurs independently of ligand stimulation [43]. However, it is clear that the formation of the Tie-1/Tie-2 complex on the cell surface modifies the Tie-2 cell signaling. Therefore, the responsiveness of endothelial cells to angiopoietins is determined by the relative levels of Tie-2 and Tie-1 [44]. Particularly, Tie-1 is considered as a Tie-2 inhibitory co-receptor that reduces Ang-1 signaling through Tie-2 [45]. Here, we have shown that UT7/mpl cells express Tie-1, whose level is upmodulated by TPO; this finding may have potentially important implications for the control of Tie-2 signaling during various stages of megakaryocytic differentiation.

In conclusion, our results support the existence of an angiopoietin/Tie-2 autocrine loop operating in megakaryocytic cells and controlling the proliferation/differentiation of these cells in cooperation with TPO.
